# Supplementary material for: The steroid-inducible pOp6/LhGR gene expression system is fast, sensitive and does not cause plant growth defects in rice (Oryza sativa)
Source: BMC Plant Biol. 2021 Oct 9;21:461. doi: 10.1186/s12870-021-03241-w (PMC8501728; doi:10.1186/s12870-021-03241-w)
Supplement: Supplementary file 1 — Additional file 1: Supplementary Figure S1. Cloning strategy for preparation of the pVecLhGR2 construct. [file 12870_2021_3241_MOESM1_ESM.docx]

**SUPPLEMENTARY FIGURE**

**
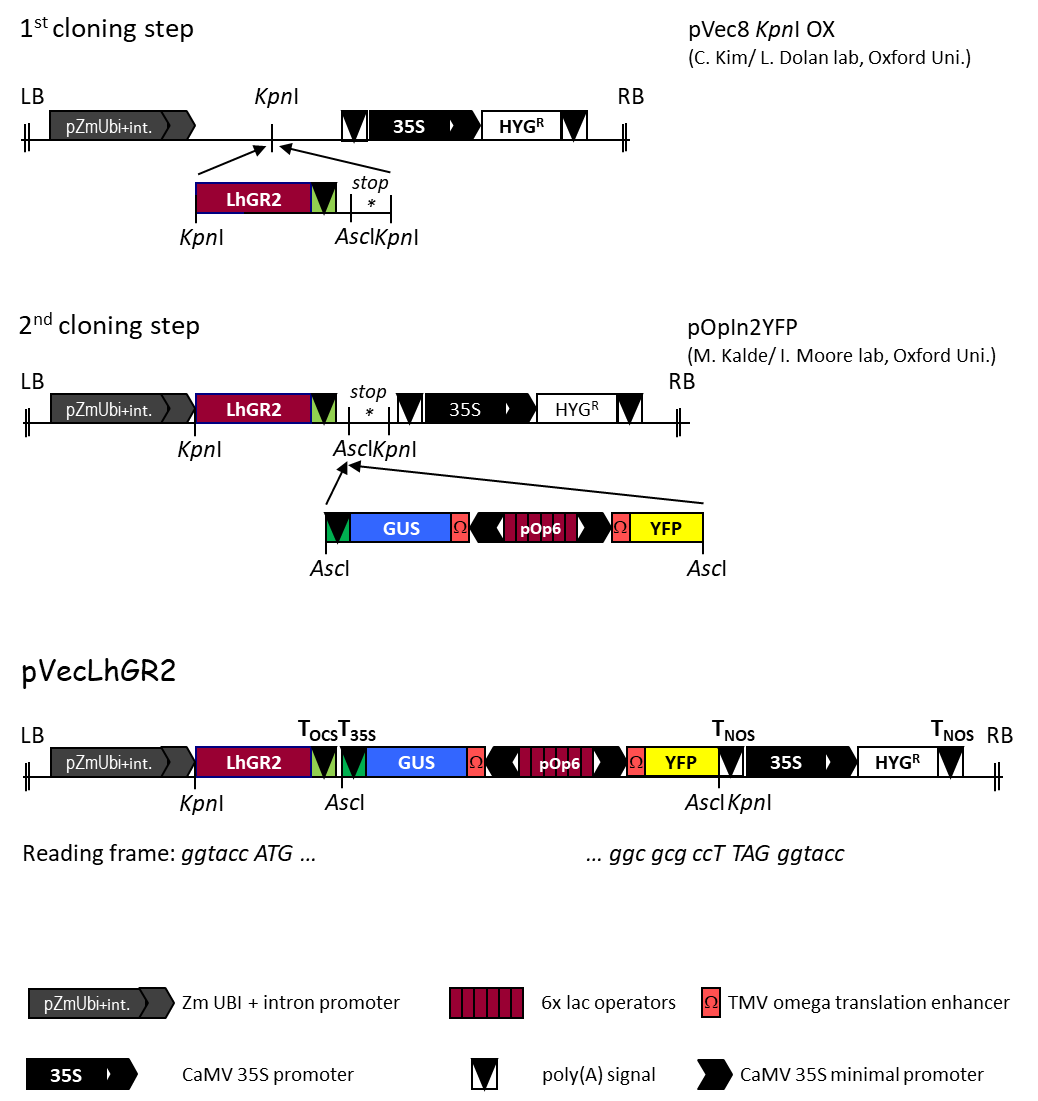
**

**Supplementary Figure S1: Cloning strategy for preparation of the pVecLhGR2 construct**
